# Low Computational Signal Acquisition for GNSS Receivers Using a Resampling Strategy and Variable Circular Correlation Time

**DOI:** 10.3390/s18020678

**Published:** 2018-02-24

**Authors:** Yeqing Zhang, Meiling Wang, Yafeng Li

**Affiliations:** School of Automation, Beijing Institute of Technology, Beijing 100081, China; wangml@bit.edu.cn (M.W.); 3120140372@bit.edu.cn (Y.L.)

**Keywords:** signal acquisition, bandpass sampling, circular correlation time, computational complexity, time consumption, GNSS receivers

## Abstract

For the objective of essentially decreasing computational complexity and time consumption of signal acquisition, this paper explores a resampling strategy and variable circular correlation time strategy specific to broadband multi-frequency GNSS receivers. In broadband GNSS receivers, the resampling strategy is established to work on conventional acquisition algorithms by resampling the main lobe of received broadband signals with a much lower frequency. Variable circular correlation time is designed to adapt to different signal strength conditions and thereby increase the operation flexibility of GNSS signal acquisition. The acquisition threshold is defined as the ratio of the highest and second highest correlation results in the search space of carrier frequency and code phase. Moreover, computational complexity of signal acquisition is formulated by amounts of multiplication and summation operations in the acquisition process. Comparative experiments and performance analysis are conducted on four sets of real GPS L2C signals with different sampling frequencies. The results indicate that the resampling strategy can effectively decrease computation and time cost by nearly 90–94% with just slight loss of acquisition sensitivity. With circular correlation time varying from 10 ms to 20 ms, the time cost of signal acquisition has increased by about 2.7–5.6% per millisecond, with most satellites acquired successfully.

## 1. Introduction

As the most mature Global Navigation Satellite System (GNSS), Global Positioning System (GPS) has occupied the dominated position in many aspects, such as civil application, scientific research, and military defense. Further, the GPS modernization plan has drawn remarkable improvement that adds new civil signals transmitted by parts of GPS satellites for high-accuracy navigating and positioning in complex environments. These new signals possess better performance of self-/cross-correlation, as well as the ability to suppress narrowband interference and correct transmission delay of the ionosphere [1]. The longer code period of new signals, especially GPS L2C signals, indicates that computational complexity and time consumption will become greater for conventional acquisition algorithms [2]. Besides, multimode multi-frequency GNSS receivers are applied in many fields nowadays. Broadband receiving of multi-frequency signals is the popular and feasible technique to minimize the energy consumption and physical size of those receivers [3,4]. However, broadband receiving requires higher sampling frequency, which dramatically increases computation and time consumption of the subsequent signal processing. It brings an inevitable dilemma for signal acquisition.

In principle, signal acquisition is the chief operation to identify the visibility of satellites and provide estimated values of carrier frequency and code phase of received signals. Based on these estimated values, signal tracking can be successfully activated for following baseband operations. Actually, signal acquisition is a two-dimensional search process over carrier frequency and code phase uncertainty [5], by correlating with local replicated ones. Successful acquisitions are affirmed if acquisition decision statistics exceed the acquisition threshold. Conceivably, signal acquisition is quite time-consuming and requires tremendous computation [6]. Meanwhile, computation acts as the bottleneck of GNSS receiver applications, as it determines the lowest sensitivity of the baseband processing. Therefore, requirements for faster, less computationally demanding, and more efficient acquisition approaches are put forward to GNSS receivers in an extensive research field [1,5,7].

Attributed to the primary convolution and correlation operations in the acquisition process, various sophisticated acquisition algorithms have been investigated to enhance the efficiency of Fast Fourier Transform (FFT) to confront the challenge of computation and time cost. For instance, ideas of sparse FFT [8], fast FFT [9], reduced-size FFT [10], and averaging correlation [11] are attempted to reduce computational complexity when acquiring satellite signals. Specific to *n*-point FFT operations of parallel search acquisition algorithms, various divisions have presented their respective effects on relieving the burden of computation and time cost, such as radix-2 and radix-4 FFT [12] or by replacing with *m* sets *2k*-point FFT [13]. Besides, multi-step acquisition methods [14,15,16,17,18] also are studied to seek for better performance of signal acquisition on computation and time consumption. Taking advantage of the double-channel structure of modernized GPS signals, the joint data-pilot channel strategy [19] outperforms other individual channel strategies on signal acquisition. In fact, computational complexity and time consumption of signal acquisition are highly dependent on the data size of sampled signals used for convolution and correlation operations. Although extensive study has been conducted, most of them concentrate on reducing instant processing points of FFT. Only a few investigations [20,21,22] have carried out attempts to directly shorten or down-sample the received original signals. The bandpass sampling theory [23], which is a direct expansion of the traditional Shannon sampling theorem, has been widely applied in the fields of communication and radar signal processing [24,25,26]. In the GNSS community, the bandpass sampling theory was adopted to design a direct conversion of GNSS carrier signal [27,28,29,30] in order to reduce the sampling frequency of the direct RF sampling front end of GNSS receivers. However, currently mainstream commercial GNSS receivers generally employ the intermediate frequency (IF) sampling front end. In the design of IF sampling front end for multi-frequency GNSS receivers, its bandwidth is generally much larger than that of the single-frequency front end, in order to incorporate multiple signals located at adjacent frequency points in one channel of the IF front end. Owing to the fact that signals of each frequency point are acquired separately, signal acquisition can be realized within the lower bandwidth. It exactly inspires us to apply the bandpass sampling theory to the efficient and fast signal acquisition of the IF sampling GNSS receivers.

As another important solution contributing to the signal acquisition challenge, massive correlation-related approaches also have been widely investigated. From the perspective of correlation period, Qaisar et al. [31] introduced an interesting chip-wise correlation strategy that accumulated code samples in one period and calculated correlation values for each chip period to reduce the search space and computation. In contrast, Zhu et al. [32] extended the correlation time to an entire duration of navigation data bit, aiming at enhancing the sensitivity of receivers. Further, Zhu et al. [33] proposed a variable time parallel acquisition scheme and different accumulation schemes over multiple code periods to detect weak signals. Jin et al. [34] exploited a fusion acquisition method of multiple correlation strategies to reduce correlation loss caused by sign transition of high data rate signals. Especially, a small increase of computation emerged using the delay-accumulation method [35] and data-pilot optimized combining acquisition methods [36]. Additionally, Ta et al. [37] presented a partial acquisition algorithm using specially-designed matched filters and differential post-correlation techniques to simplify computational complexity of signal acquisition. Although increasing correlation time is an effective way to ensure acquisition accuracy, computation and time consumption generally present higher expense due to the longer correlation time. Further, it is difficult to find out many researches that have done detailed exploration on the relationship between acquisition performance and correlation time, and that have been applied effectively to signal acquisition of GNSS receivers.

With regard to broadband multi-frequency GNSS receivers, we propose a resampling strategy and variable circular correlation time in this paper to decrease computational complexity and time consumption of signal acquisition. Inspired by the bandpass sampling theory, the resampling strategy reduces greatly the data size of the sampled signal with just slight loss of signal quality. It lays the computational foundation for FFT, correlation and convolution operations of signal acquisition. In particular, the resampling strategy is implemented step-by-step to demonstrate the capability of combining with existing FFT-/correlation-based acquisition algorithms. Taking the longer code period into consideration, the relationship between circular correlation time and acquisition performance is concerned for the potential of shortening circular correlation time to adapt to different signal strength conditions and thereby increase the operation flexibility of GNSS signal acquisition. Moreover, the acquisition threshold is defined as the ratio of the highest and second highest correlation results in the search space of carrier frequency and code phase. Computational complexity is formulated by amounts of multiplication and summation operations in the signal acquisition.

This paper is organized as follows: Section 2 introduces the characteristics of the GPS L2C signal as an example, and the framework of GNSS receivers to indicate the crucial role of the proposed strategies. Next, Section 3 elaborates on the proposed resampling strategy and variable circular correlation time, in terms of the principle, realization, coarse and fine acquisition, as well as performance evaluation. Comparative experiments and performance analysis are performed on four sets of real GNSS signals in Section 4, to verify the effectiveness of the resampling strategy and variable circular correlation time. Conclusions are drawn in Section 5.

## 2. Characteristics of Satellite Signals and Framework of GNSS Receivers

### 2.1. Characteristics of Satellite Signals

In this paper, we take the GPS L2C signal as an example of GNSS signals. GPS L2C signal is the civil signal, newly added on the existing L2 frequency band and modulated by civil-moderate (CM) and civil-long (CL) codes. The mechanism of the GPS L2C signal is described as follows: Firstly, the navigation message is encoded by forward error correction (FEC) coder and modulated on CM code to comprise the data channel. Meanwhile, without navigation message, CL code acts as the pilot channel by direct sequence spread spectrum (DSSS) method. After that, signals of data and pilot channels are separately added on the baseband signal by time division multiplexing (TDM) method, and further modulated by binary phase shift keying (BPSK) method on carrier wave. Finally, the generated signal is transmitted by satellites. GPS L2C signal received from satellites can be modeled as [4,22]:

(1) $$S_{L2}\left(t\right)=A\left[D\left(t-\theta \right)C_{M}\left(t-\theta +kT_{M}\right)+C_{L}\left(t-\theta \right)\right]cos\left[2\pi \left(f_{IF}+f_{D}\right)t+\varphi \right]+n\left(t\right)$$

where A is the signal amplitude and D(t) is the navigation message. $C_{M}\left(t\right)$ and $C_{L}\left(t\right)\;$ represent CM and CL codes, respectively. θ is code phase in the received signal. $T_{M}$ denotes the period of CM code and k indicates the number of CM epochs in the current CL epoch, 0≤k≤74. The carrier frequency of the received signal consists of the intermediate frequency $f_{IF}$ and Doppler frequency shift $f_{D}$, which results from the relative motion between the satellite and the receiver. φ refers to the initial carrier phase. Besides, n(t) is the thermal noise in the received signal, denoted as the additive white Gaussian noise. It is noticeable that the signal is just composed of the cosine term on account of the BPSK method.

### 2.2. Framework of GNSS Receivers

**Figure 1:**
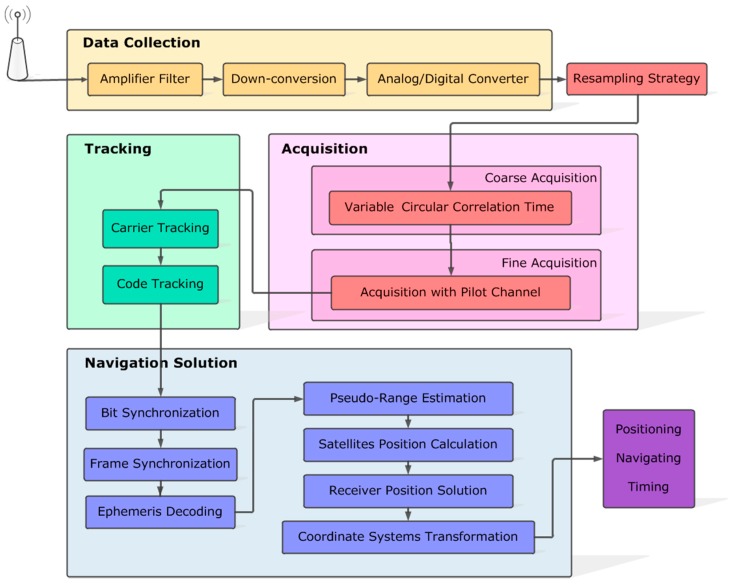
The signal processing framework of GNSS receivers. The modules marked as red are the improved strategies of this paper, including the resampling strategy, variable circular correlation time and acquisition with pilot channel.

Primarily, the signal processing framework of GNSS receivers mainly includes 4 parts: data collection, signal acquisition, signal tracking, and navigation solution, as shown in Figure 1. Firstly, the original signal received by the antenna is converted from low-power high-frequency into intermediate-frequency, and then sampled as digital to send to different channels of receivers. Next, visible satellites are identified by signal acquisition. It is also obtained that estimated values of carrier frequency and code phase of acquired satellite signals. Then, based on these estimated values, carrier wave and pseudo-random noise (PRN) code are accurately removed from the received signal by applying phase locked loop (PLL) and delay locked loop (DLL) to signal tracking. Finally, the navigation message is extracted by bit and frame synchronization to calculate position and velocity of the receiver. In this way, satellite signal has been processed by GNSS receivers to accomplish the functions of positioning, navigating, and timing.

In this paper, the resampling strategy is proposed to decrease the data size of the sampled signal without obvious loss of signal quality, so as to greatly reduce computational complexity and time consumption of signal acquisition. Currently, signal acquisition is fundamentally realized by the serial search algorithm, parallel frequency space search algorithm, and parallel code phase search algorithm. By contrast, the parallel code phase search algorithm is the most efficient one that searches all code phases at the same time for each frequency bin. Hence, we chose the parallel code phase search algorithm on behalf of conventional acquisition algorithms to perform the resampling strategy. Besides, signal acquisition is improved by variable circular correlation time.

## 3. Methodology of the Resampling Strategy and Variable Circular Correlation Time

### 3.1. Principle of the Resampling Strategy

Generally, the original signal can be reconstructed from the sampled signal without loss of signal quality if the sampling frequency is higher than twice the upper cutoff frequency of the original signal. However, this principle is not suitable for signal acquisition of multimode multiband GNSS receivers. These receivers can receive multi-frequency satellite signals at the same time. Therefore, a much higher sampling frequency is required to sample the received broadband signal. Obviously, it is rather aimless, low-efficiency, and of low quality to acquire a specific frequency signal by sampling the received broadband signal in this way. Moreover, the higher sampling frequency results in much more computation and time consumption for signal acquisition, because the data size of the sampled signal is much larger. To address this dilemma of balance between the sampling frequency and the greatly increasing computation, we propose the resampling strategy based on the bandpass sampling theory [23].

**Theorem** **1.** *Bandpass Sampling Theory: Assume that the frequency band of a time*-*continuous signal* x(t) *is located at* $\left[-f_{u},-f_{l}]\;\cup^{}\;[f_{l},\;f_{u}\right]$*, where* $f_{l}$ *and* $f_{u}$ *are the lower and upper cutoff frequencies, respectively.* B *denotes the bandwidth of the signal* x(t)*,* $B=f_{u}-f_{l}$*. In order to reconstruct the original signal* x(t) *without spectrum aliasing from the sampled sequence* X[k]*, the acceptable sampling frequency* $f_{s}$ *should be in the range of*

(2) $$\frac{2f_{u}}{n}\leq f_{s}\leq \frac{2f_{l}}{n-1},\;n\in \left[1,\;N\right]$$

*where* $N=Z^{-}\left(f_{u}/B\right)$ *denotes the maximum integer no more than* $f_{u}/B$*. Note that,* n=1 *just makes sense for the case that* $f_{s}\in \left[2f_{u},\;+\infty \right)$*.*

**Figure 2:**
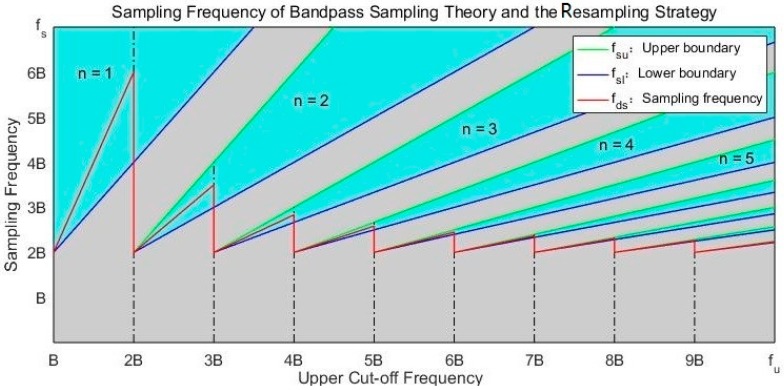
Acceptable sampling frequency (cyan areas) based on the bandpass sampling theory. Blue and green lines are lower and upper boundaries of the acceptable sampling frequency. The red line indicates the resampling frequency of the proposed strategy.

The relationship between $f_{s}$ and $f_{u}$ is illustrated in Figure 2. In order to avoid spectrum aliasing when reconstructing the original signal, the sampling frequency $f_{s}$ should be chosen from cyan areas, not gray ones. In Figure 2, the available range of the sampling frequency $f_{s}$ consists of N non-overlapping cyan sections, denoting as $\Phi_{n}=\left[2f_{u}/n,\;2f_{l}/\left(n-1\right)\right],\;\left(n=1,2,\ldots ,N\right)$ and $f_{s}\in \cup_{n=1}^{N}\Phi_{n}$. It is realizable for the sampling frequency to be much lower, such as [2B, 4B], instead of the conventional range ($f_{s}\geq 2f_{u},$ the cyan n=1). The parameters, $f_{su}$, $f_{sl}$, and $f_{ds}$ will be discussed for the proposed resampling strategy later.

### 3.2. Realization of the Resampling Strategy for Signal Aquisition

Sampling the time-continuous signal s(t) with the frequency $f_{s}$, the frequency spectrum of the sampled sequence $S\left(kT_{s}\right)$ is the same as that of the original signal s(t) in the frequency domain, periodically extended with the period $f_{s}$. Hence, with the appropriate sampling frequency, expanding components of the sampled sequence will not fold with the frequency spectrum of the original signal in $\left[-f_{u},-f_{l}]\;\cup^{}\;[f_{l},\;f_{u}\right]$. In this way, the original signal can be recovered from the sampled sequence without loss of signal quality. Based on the bandpass sampling theory, the proposed resampling strategy is realized as Algorithm 1 for signal acquisition of GNSS receivers.

**Algorithm 1 Realization of the Resampling Strategy for GNSS Signal Acquisition****Input:**
The received broadband satellite signal;
**Output:**
Doppler frequency and code phase offset of the received satellite signal.Filter out side lobes of the original broadband satellite signal;Update the parameters of the resampling strategy;Resample the main lobe signal with the resampling frequency;Restore the resampling acquisition results to that of conventional acquisition algorithms;**Return**: the acquired Doppler frequency and code phase offset of the received satellite signal.

Note the broadband signal received by multimode multiband GNSS receivers as $\bar{S}\left(t\right)=\left\{S_{L1}\left(t\right),\;S_{L2}\left(t\right),\;S_{L5}\left(t\right),\;S_{E5}\left(t\right),\;\ldots \right\}$. It includes GPS L1, L2, L5 signals, as well as signals transmitted by other satellite systems, such as Galileo, GLONASS, and BeiDou. We intend to assume that the main lobe refers to the GPS L2C signal, and other signals are regarded as the side lobe signals. A bandpass filter is designed to extract the main lobe signal $S_{L2}\left(t\right)$ from the broadband signal $\bar{S}\left(t\right)$.

(3) $$\{\begin{array}{c} f_{l}=\;f_{IF}-\frac{B}{2}\;\\ f_{u}=\;f_{IF}+\frac{B}{2}\;\\ \omega_{p}=\left[\frac{2f_{l}}{f_{s}}-\Delta \omega_{f}\;\frac{2f_{u}}{f_{s}}+\Delta \omega_{f}\right]\; \end{array}$$

where $f_{l}$ and $f_{u}$ are the lower and upper cutoff frequencies of the main lobe signal $S_{L2}\left(t\right)$. $f_{s}$ is the conventional sampling frequency for the broadband signal $\bar{S}\left(t\right)$. $f_{IF}$ and B represent the intermediate frequency and bandwidth of $S_{L2}\left(t\right)$. It is guaranteed that $f_{s}\gg 2f_{u}$ in order to effectively sample $S_{L2}\left(t\right)$. The bandpass filter is designed as an n-order Hamming-window linear-phase finite impulse response (FIR) filter. The pass band $\omega_{p}$ is the vector of the normalized cutoff frequencies. $\Delta \omega_{f}$ is the margin to ensure the main lobe signal reserved completely.

By employing the designed bandpass filter on the broadband signal, the side lobe signals are filtered out while the main lobe remains. This plays as a foundational block to avoid frequency aliasing and reduce the data size of the sampled signal.

(1) Update the Parameters of the Resampling Strategy

A resampling filter is designed to resample the main lobe signal $S_{L2}\left(t\right),$ obtained by the bandpass filter. The lower and upper cutoff frequencies and bandwidth of the resampling filter, $f_{dl},\;f_{du}$ and $\;B_{d}$, are expressed as

(4) $$\{\begin{array}{c} f_{dl}=\;f_{l}\;\\ f_{du}=\;f_{u}\;\\ B_{d}=f_{du}-f_{dl}\; \end{array}$$

Based on the bandpass sampling theory in Theorem 1, the lower and upper boundaries of the acceptable sampling frequency, $f_{sl}$ and $f_{su}$, are determined as

(5) $$f_{sl}=\;\{\begin{array}{c} 2f_{du},\;\;\;\;\;\;\;\;\;\;\;\;\;if\;n<1\\ \frac{2f_{du}}{n},\;\;\;\;\;\;\;\;\;\;\;\;\;if\;n\geq 1 \end{array}$$

(6) $$f_{su}=\;\{\begin{array}{c} f_{sl},\;\;\;\;\;\;\;\;\;\;\;\;\;if\;n\leq 1\\ \frac{2f_{dl}}{n-1},\;\;\;\;\;\;\;\;\;\;\;if\;n>1 \end{array}$$

Here, n=1,2,…,N. And $N=Z^{-}\left(f_{du}/B_{d}\right)$. The center of the acceptable sampling frequency range $\left[f_{sl},\;f_{su}\right]$ is regard as the resampling frequency $f_{ds}$:

(7) $$f_{ds}\;=\;\frac{f_{sl}+f_{su}}{2}$$

The parameters of the resampling strategy, $f_{sl},\;f_{su}$ and $f_{ds}$, are illustrated as the blue, red and green curves in Figure 2, respectively. The number n is chosen as the maximum integer N to decrease the resampling frequency as much as possible. For that $S_{L2}\left(t\right)$ is the high-frequency narrowband signal, the conventional sampling frequency $f_{s}$ is much higher than the bandwidth ($f_{s}\geq 2f_{u}\gg B$). As the red curve in Figure 2, the resampling frequency $f_{ds}$ is maintained in the lower frequency range $\left[2B_{d},\;6B_{d}\right],\;\left(B_{d}\ll f_{u}\right)$.

(2) Resample the Main Lobe Signal with the Resampling Strategy

When applying the resampling filter to the main lobe signal $S_{L2}\left(t\right)$, the sampling frequency and intermediate frequency are updated to $f_{ds}$ and $f_{dIF}.$ Especially, $f_{ds}$ is significantly decreased compared to the conventional one $f_{s}$. The original intermediate frequency $f_{IF}$ also is equivalent to the much lower one $f_{dIF}$, which is the remainder of $f_{IF}$ and $f_{ds}$, as Equation (8). The resampled sequence $S_{d}\left(k\right)$ is expressed as Equation (9).

(8) $$f_{dIF}\;=\;ℳ\left(f_{IF},\;f_{ds}\right).$$

(9) $$\{\begin{array}{c} S_{d}\left(k\right)\;=\;S_{s}\left(k\right)\\ k=Z^{+}\left(\frac{i\times f_{s}}{f_{ds}}\right) \end{array}$$

(10) $$L_{d}\;=\;Z^{-}\left(\frac{L\times f_{ds}}{f_{s}}\right)$$

where $Z^{+}\left(x\right)$ denotes the minimum integer bigger than x. $S_{s}\left(\cdot \right)$ and $S_{d}\left(\cdot \right)$ are the sequences obtained by sampling $S_{L2}\left(t\right)$ with $f_{s}$ and $f_{ds}$, respectively. i is the index of samples in the sequence $S_{s}\left(i\right)$, i=1,2,…,L. L denotes the length of $S_{s}\left(i\right)$, while k is that of the resampled sequence $S_{d}\left(k\right),\;k=1,2,\ldots ,L_{d}$. Due to $f_{ds}\ll f_{s}$, $L_{d}$ is much shorter than $L\;\left(L_{d}\ll L\right)$.

Therefore, $S_{d}\left(k\right)$ is extracted from $S_{s}\left(i\right)$ to equivalently express the main lobe signal $S_{L2}\left(t\right)$. Though the length is greatly reduced, $S_{d}\left(k\right)$ is sufficient to reconstruct the main lobe signal. It is beneficial for reducing the computation and time consumption of signal acquisition.

(3) Restore the Resampling Acquisition Results to that of Conventional Acquisition Algorithms

The parameters of the resampling strategy, $f_{ds}$ and $f_{dIF}$, and the resampled sequence $S_{d}\left(k\right)$ are utilized to obtain the acquisition results, including actual carrier frequency $f_{dc}$, Doppler frequency shift $f_{dD}$, and code phase offset $\theta_{d}$. In order to be applied to signal tracking in the conventional way, the acquisition results of the resampling strategy are restored equivalently to the carrier frequency $f_{c}$ and code phase offset θ of the conventional acquisition algorithm. In this way, the capability of the proposed resampling strategy to combine with conventional acquisition algorithms has been proven.

(11) $$f_{dD}=\;f_{dc}-f_{dIF}\;$$

(12) $$f_{c}\;=\;f_{dD}\;+\;f_{IF}$$

(13) $$\theta =\;Z^{-}\left(\frac{\theta_{d}\times f_{s}}{f_{ds}}\right)\;+1$$

**Figure 3:**
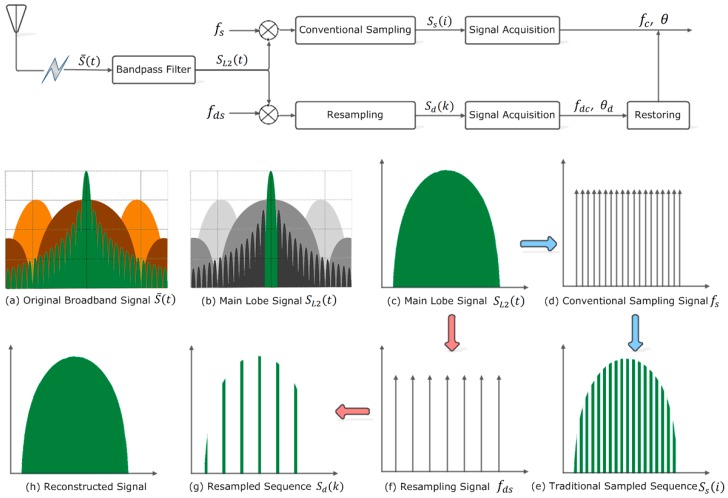
Signal flow chart by applying the resampling strategy and the convolutional method to the received broadband satellite signal. The green, dark brown, and orange represent frequency spectra of GPS L2C, P(Y), and M code signals, respectively.

With the resampling strategy acting on the original broadband signal, the signal flow chart is illustrated in Figure 3. Remarkably, the data size of the resampled signal is decreased from L to $L_{d},\;\left(L_{d}\ll L\right)$. Therefore, the resampling strategy is capable to greatly reduce computation and time consumption of signal acquisition.

### 3.3. Coarse Acquisition with Variable Circular Correlation Time

Conventional acquisition methods usually set the circular correlation time as one period of PRN code. Those are low-efficiency, time-consuming, and of large computation. Especially, code periods of GPS L2C signal are as long as 20 ms for CM code and 1.5 s for CL code. Actually, the shorter circular correlation time could quicken signal acquisition if the received GPS signal is strong enough. For weak signals in noisy environments, the longer circular correlation time ensures the acquisition performance. For the purpose of exploring the effects of different circular correlation times on the performance of signal acquisition, we introduce variable circular correlation time in the coarse acquisition.

The reference signal $\;S_{r}\left(i\right)$ of length $L_{r}$ is extracted from the resampled signal $S_{d}\left(i\right)$. The zero-padding PRN code $C_{r}\left(i\right)$ is introduced to calculate circular correlation with the reference signal to obtain accurate carrier frequency and code phase.

(14) $$\{\begin{array}{c} P_{r}=\;P_{c}+P_{x}\\ L_{r}=\;L_{c}+L_{x} \end{array},\;P_{x}\in \left(0,\;P_{c}\right]$$

(15) $$S_{r}\left(i\right)\;=\;S_{d}\left(i\right),\;i=1,2,\;\ldots ,\;L_{r}.$$

(16) $$C_{r}\left(i\right)\;=\;\{\;\begin{array}{c} C_{M}\left(i\right),\;i=1,\;2,\;\ldots ,\;L_{x}\;\;\;\;\;\;\;\;\;\;\;\;\;\;\;\;\;\;\;\;\;\;\\ 0,\;i=L_{x}+1,\;L_{x}+2,\;\ldots ,\;L_{x}+L_{c}. \end{array}$$

Specifically, $P_{c}$ is the period of PRN code and $P_{x}$ represents variable circular correlation time, $P_{x}\in \left(0,\;P_{c}\right]$. If $P_{x}$ = $P_{c}$, the variable circular correlation time method is the same as conventional acquisition algorithms. $L_{r},\;L_{c}$ and $L_{x}$ are the lengths of signals with periods $P_{r},\;P_{c}$, and $P_{x}$.

At frequency search step k, the reference signal $S_{r}\left(i\right)$ is multiplied with local carrier replica $W_{l}\left(k\right)$ of frequency f(k) to remove carrier wave from $S_{r}\left(i\right)$ and obtain the baseband signal $S_{w}\left(k\right)$

(17) $$W_{l}\left(k\right)=e^{j2\pi f\left(k\right)}\;$$

(18) $$S_{w}\left(k\right)=\;W_{l}\left(k\right)\cdot S_{r}\left(i\right)$$

**Figure 4:**
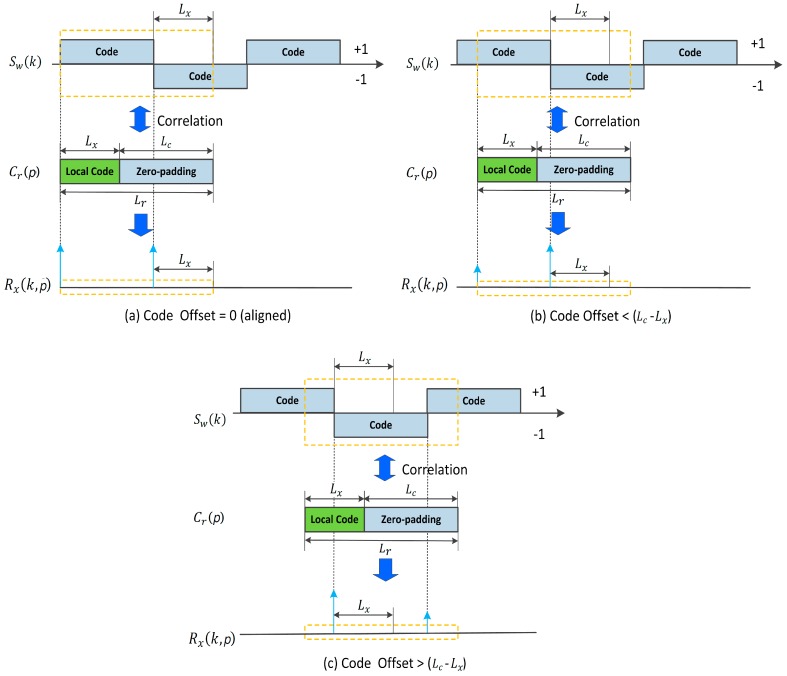
Circular correlation results of the baseband signal $S_{w}\left(k\right)$ and the local zero-padding code $C_{r}\left(p\right)$: (**a**) code offset between $S_{w}\left(k\right)$ and $C_{r}\left(p\right)$ is 0 (aligned); (**b**) code offset between $S_{w}\left(k\right)$ and $C_{r}\left(p\right)$ is less than $\left(L_{c}-L_{x}\right)$ samples; (**c**) code offset between $S_{w}\left(k\right)$ and $C_{r}\left(p\right)$ is more than $\left(L_{c}-L_{x}\right)$ samples.

With different code phase offsets, the baseband signal $S_{w}\left(k\right)$ is circular correlated with the local zero-padding code $C_{r}\left(p\right),$ as illustrated in Figure 4. $r_{x}\left(k,\;p\right)$ is the circular correlation result of $S_{w}\left(k\right)$ and $C_{r}\left(p\right)$. FFT is adopted to quicken the correlation calculation in the time domain, as Equation (20). $ℱ{\left[x\right]}^{*}$ refers to the complex conjugate value of ℱ[x].

(19) $$r_{x}\left(k,p\right)=\overset{L_{r}}{\underset{i=1}{\sum }}S_{w}\left(k,i\right)C_{r}\left(p+i\right),\;p=1,\;2,\;\ldots ,\;L_{r}.$$

(20) $$ℱ[r_{x}\left(k,\;p\right)]\left(n\right)=\overset{L_{r}}{\underset{p=1}{\sum }}\overset{L_{r}}{\underset{i=1}{\sum }}S_{w}\left(k,i\right)C_{r}\left(p+i\right)e^{\frac{-j2\pi np}{L_{r}}}\;=\;ℱ[S_{w}\left(k\right)]\cdot ℱ{\left[C_{r}\left(p\right)\right]}^{*}$$

Correlation magnitude reflects the self-/cross-correlation relationship of the received satellite signal and the local code replica. When they are completely aligned, a much higher correlation peak is gained. In the search space of carrier frequency and code phase, the correlation magnitude $M_{x}\left(k,p\right)$ is formulated as

(21) $$M_{x}\left(k,\;p\right)=\left|r_{x}\left(k,p\right)\right|=\sqrt{R^{2}\left[r_{x}\left(k,p\right)\right]+I^{2}\left[r_{x}\left(k,\;p\right)\right]}$$

where R(·) and I(·) denote the real and imaginary parts of a complex data. By Equations (19)–(21), it is very easy to find out the highest correlation peak in the search space. Denote the corresponding carrier frequency and code phase as ${\hat{f}}_{dc}$ and ${\hat{\theta }}_{d}$, respectively.

In this paper, acquisition threshold is defined as the ratio of the highest and second highest correlation values in the search space of carrier frequency and code phase. The second highest peak is found out in the neighborhood range of the highest one. The left neighborhood range is obtained as $\left[0,\;{\hat{\theta }}_{d}\right]\cap^{}\left[\theta_{l2},\;\theta_{l1}\right]$ and the right is $\left[\theta_{r1},\;\theta_{r2}\right]\cap^{}\left[{\hat{\theta }}_{d},\;L_{r}\right]$.

(22) $$\{\begin{array}{c} \begin{array}{c} \theta_{l1}={\hat{\theta }}_{d}-\Delta_{\theta }\;\\ \theta_{l2}={\hat{\theta }}_{d}-L_{x}+\Delta_{\theta }\\ \theta_{r1}={\hat{\theta }}_{d}+\Delta_{\theta }\; \end{array}\\ \theta_{r2}={\hat{\theta }}_{d}+L_{x}-\Delta_{\theta } \end{array}$$

where $\Delta_{\theta }$ is the one-chip-period range, excluded from the neighborhood range, for it is too close to the highest peak to find out the useful second peak. If the correlation ratio is bigger than the acquisition threshold, it is confirmed that the corresponding satellite is visible and the signal of frequency ${\hat{f}}_{dc}$ and code phase ${\hat{\theta }}_{d}$ is acquired successfully.

Note that the frequency spectrum of $C_{r}\left(p\right)$ is asymmetrical, such that the former half part occupies most of signal power. Thus, we just use the former to compute circular correlation results. This reduces FFT samples by half, so as to decrease computation of signal acquisition. Additionally, $L_{x}$ is highly related to the amount of FFT samples for signal acquisition. Equation (19) provides a good indication to reduce computation and time cost of signal acquisition by decreasing the sampling frequency $f_{s}$ and circular correlation time $P_{x}$. That is exactly realized by the proposed resampling strategy and variable circular correlation time.

### 3.4. Fine Acquisition with Pilot Channel

For GPS L2C signals, the phase of CL code is aligned with that of the CM code. The reference signal $S_{fr}\left(i\right)$ of length $L_{x}$ is directly extracted from the main lobe signal $S_{L2}\left(t\right)$ with code phase ${\hat{\theta }}_{d}$. Similarly, the CL code is removed from $S_{fr}\left(i\right)$ by multiplying with the local CL code replica to get the baseband signal $S_{fx}\left(i\right)$. Considering that no navigation message is modulated on the signal of pilot channel, FFT acts as the frequency discriminator to find out the accurate carrier frequency.

(23) $$ℱ[S_{fx}\left(i\right)]=\overset{N_{L}}{\underset{i=1}{\sum }}S_{fx}\left(i\right)e^{\frac{-j2\pi ni}{N_{L}}}$$

where $ℱ[S_{fx}\left(i\right)]$ is the FFT result of $S_{fx}\left(i\right)$. The amount of FFT samples $N_{L}$ is set as the power of 2 closest to $L_{x}$ to speed up FFT calculation, when the signal length $L_{x}$ is not an exact power of 2. As $ℱ[S_{fx}\left(i\right)]$ is a periodic complex even signal, the frequency spectrum is symmetrical in the frequency range $\left[0,\;f_{ds}\right].$ Hence, the one-sided FFT is adopted.

Assume $M_{fx}\left(\hat{m}\right)$ as the maximum magnitude of $ℱ[S_{fx}\left(i\right)]$ in the frequency range $\left[0,\;f_{ds}\right],$ and $\hat{m}$ is the corresponding index of $ℱ[S_{fx}\left(i\right)]$. The high-accuracy carrier frequency of the received signal is discriminated as $f_{dc}$ in the fine acquisition:

(24) $$f_{dc}=\{\;\begin{array}{c} \hat{m}\;\frac{f_{ds}\;}{N_{L}},\;if\;\hat{m}\leq \frac{N_{L}\;}{2}\;\\ (N_{L}-\hat{m})\;\frac{f_{ds}\;}{N_{L}},\;if\;\hat{m}>\frac{N_{L}\;}{2}\; \end{array}$$

Note that the frequency resolution $f_{ds}/N_{L}$ is inversely proportional to $N_{L}$, and $N_{L}$ is highly dependent on $L_{x}$. From this perspective, circular correlation time is positively relative to the resolution of acquired carrier frequency. That is, the longer circular correlation time will ultimately result in higher accurate carrier frequency in the fine acquisition. Besides, circular correlation time also affects computation and time cost of the fine acquisition, for that FFT is utilized as the frequency discriminator.

### 3.5. Performance Evaluation of Signal Acquisition

For signal acquisition, time cost is usually a stochastic variable. Mean value of time cost is concerned as one of the most important measurements to assess the performance of acquisition algorithms. Computation complexity is another crucial evaluation index. Usually, convolution operation in the time domain is converted into multiplication in the frequency domain by FFT to improve the efficiency of signal acquisition algorithms. We choose amounts of multiplication and summation operations to evaluate the computational complexity of signal acquisition.

Equation (25) presents computations of FFT ($O_{FFT}$), IFFT ($O_{IFFT}$), amplitude calculation ($O_{AMP}$), vector multiplication ($O_{VM}$) and comparison ($O_{CMP}$). N refers to the length of the FFT sequence. $O^{M}$ and $O^{A}$ denote the amounts of multiplication and summation operations, respectively.

(25) $$\{\begin{array}{c} O_{FFT}\left(N\right)=O_{IFFT}\left(N\right)=O^{M}\left(\frac{N}{2}log_{2}\left(\frac{N}{2}\right)\right)+O^{A}\left(Nlog_{2}N\right)\;\\ O_{AMP}\left(N\right)=O_{\;VM}\left(N\right)=O^{M}\left(N\right)+O^{A}\left(N-1\right)\;\\ O_{CMP}\left(N\right)=O^{A}\left(N-1\right)\; \end{array}$$

Note the computations of local code replica generation and correlation ratio calculation as $O_{lc}$ and $O_{pk}$. And the computations of coarse and fine acquisition are denoted as $O_{ca}$ and $O_{fa}$, respectively. $N_{SV}$ is the number of satellites in the search list, and $N_{f}$ is the amount of carrier frequency bins.

$$O_{lc}=O_{FFT}\left(L_{r}\right)$$

$$O_{ca}=2O_{VM}\left(L_{r}\right)+O_{FFT}\left(L_{r}\right)+O_{VM}\left(L_{r}\right)+O_{IFFT}\left(L_{r}\right)+O_{AMP}\left(L_{r}\right)$$

$$O_{pk}=O_{CMP}\left(N_{f}\times L_{r}\right)+O_{CMP}\left(N_{f}\times L_{x}\right)+O^{M}\left(N_{f}\times L_{r}\right)$$

$$O_{fa}=O_{VM}\left(L_{x}\right)+O_{FFT}\left(L_{x}\right)+O_{AMP}\left(L_{x}\right)+O_{CMP}\left(L_{x}\right)$$

Therefore, the computational complexity of signal acquisition $O_{total}$ is formulated as

(26) $$O_{total}=\left(O_{lc}+N_{f}O_{ca}+O_{pk}+O_{fa}\right)N_{SV}=O_{total}^{M}\;+\;O_{total}^{A}$$

in which,

(27) $$O_{total}^{M}=\left[\left(2N_{f}+\frac{3}{2}\right)L_{x}\log_{2}L_{x}+10N_{f}L_{x}+\frac{3}{2}L_{x}\right]N_{SV}$$

(28) $$O_{total}^{A}=\left[\left(4N_{f}+3\right)L_{x}\log_{2}L_{x}+15N_{f}L_{x}+5L_{x}-4N_{f}-5\right]N_{SV}$$

## 4. Experiments and Discussion

### 4.1. Experimental Platform and Datasets Description

The resampling strategy and variable circular correlation time proposed in this paper are realized by MATLAB software, configured with Intel dual-core 3.5 GHz CPU of i7−5930K and 16.0 G RAM. The GNSS receiver is equipped with NT1065 “Nomada” and bladeRF as the RF front end. They can simultaneously receive various GNSS satellite signals, including GPS (L1, L2, L3, L5), GLONASS (E1, E5a, E5b, E6), BeiDou (B1, B2, B3), Galileo, IRNSS, QZSS. For comparison, experiments and performance analysis are conducted on 4 sets real GNSS signals collected at the same observation station. Different configurations are adopted, such as set-up status of the resampling strategy (on/off) and circular correlation time (10–20 ms). Table 1 presents the data type, intermediate frequency, and conventional sampling frequency of experimental datasets.

Besides, basic parameters of the experimental GNSS signals are provided here: the bandwidth of main lobe is 2.046 MHz, the basis carrier frequency is 1227.6 MHz, the code frequency is 511.5 KHz, the range of frequency search band is ±5 KHz, and the bandwidth of frequency bins is 100 Hz. Acquisition threshold is set as correlation ratio $R_{c}=1.5$.

### 4.2. Performance Analysis of the Resampling Strategy

With emphasis on effects of the proposed strategy on signal acquisition, we execute the resampling strategy and the conventional algorithm (without the resampling strategy) on all datasets. Experimental results are discussed in terms of acquisition effectiveness, sensitivity, computation and time cost.

**Figure 5:**
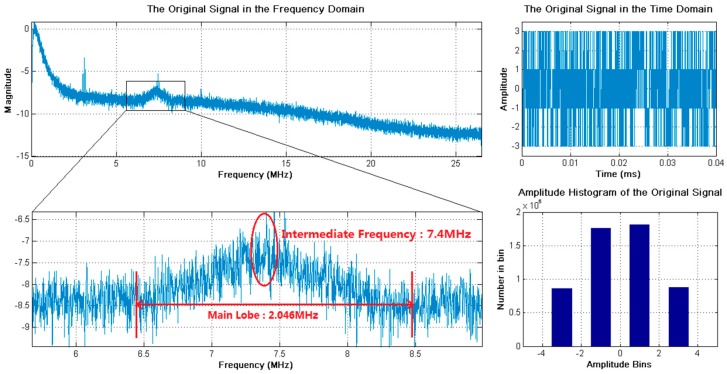
The received broadband signal of Dataset 1 in the frequency and time domains, and the amplitude distribution. The bandwidth of the main lobe signal is 2.046 MHz and the intermediate frequency is 7.4 MHz.

**Figure 6:**
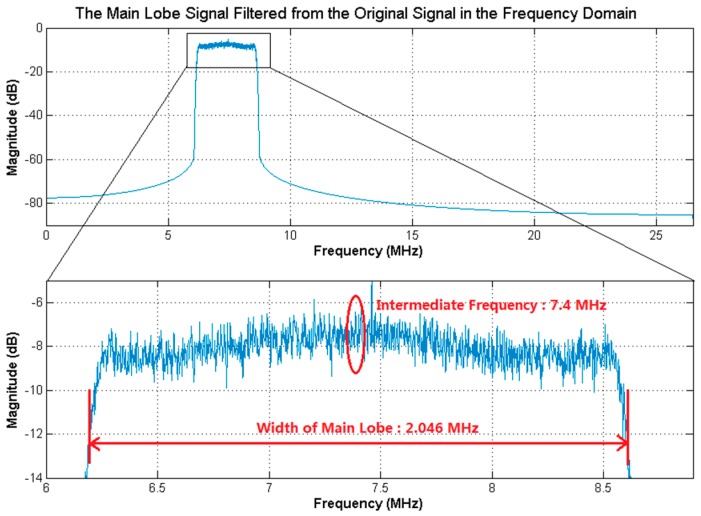
The main lobe signal filtered from the received broadband signal of Dataset 1 in the frequency domain. The bandwidth of the main lobe signal is 2.046 MHz, the intermediate frequency is 7.4 MHz and the sampling frequency is 53 MHz.

**Figure 7:**
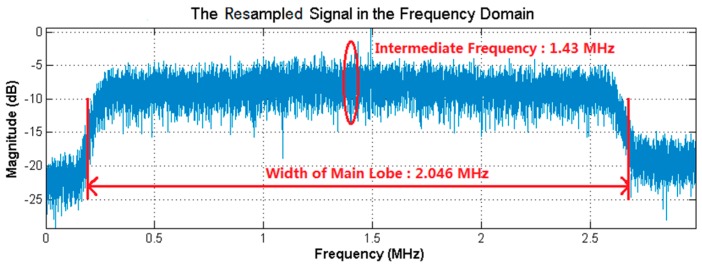
The resampled signal by applying the resampling strategy to the main lobe signal of Dataset 1 in the frequency domain. The bandwidth of the resampled signal is 2.046 MHz, the intermediate frequency is 1.43 MHz and the sampling frequency is 5.97 MHz.

Figure 5 presents the characteristics of the received broadband signal in the frequency and time domains, as well as the amplitude distribution of the received signal in Dataset 1. By applying the bandpass filter to the received broadband signal, the power of the side lobe signals was filtered out and the main lobe signal was retained, as shown in Figure 6. The bandwidth and intermediate frequency of the main lobe signal were the same as that of the received broadband signal, $B_{d}=2.046\;\mathrm{MHz}$ and $f_{IF}=7.4\;\mathrm{MHz}$. Besides, the sampling frequency of the main lobe signal was the conventional one, $f_{s}=53\;\mathrm{MHz}$. Applying the resampling strategy to the main lobe signal, the resampled signal was obtained, as presented in Figure 7. Specially, the resampled signal had the same bandwidth of the main lobe signal, while the intermediate frequency changed from $f_{IF}=7.4\;\mathrm{MHz}$ to $f_{dIF}=1.43\;\mathrm{MHz}$. Meanwhile, as a result of the resampling strategy, the sampling frequency was greatly reduced from the conventional one $f_{s}=53\;\mathrm{MHz}$ to $f_{ds}=5.97\;\mathrm{MHz}$, to reduce the data size of the resampled sequences used in FFT, IFFT, and correlation calculation. Here, circular correlation time of signal acquisition was fixed as $L_{x}=20\;\mathrm{ms}$.

The acquisition results of Dataset 1 are listed in Table 2. There were 7 satellites (PRN 5, 6, 12, 17, 24, 25, and 29) acquired successfully. It also has proved the capability of the proposed strategy to adapt to the signals with different carrier-to-noise ratios. Comparing experimental results of the resampling strategy and the conventional method (without the resampling strategy), it can be found that the two algorithms demonstrated almost the same acquisition performances, including acquired satellites, carrier frequency, Doppler frequency shift, and code phase offset. There is no obvious loss of acquisition performance for the resampling strategy. In addition, it is remarkable that the correlation magnitude of each acquired satellite decreased to about 10% as a result of the resampling strategy, shown in the bold columns in Table 2. This can be qualitatively attributed to the reduced-size samples for circular correlation by the resampling strategy, see Equation (10).

Moreover, the correlation ratio of signal acquisition reflects the relationship of the satellite signal and local PRN replica, acting as the acquisition threshold in this paper. From the bold rows listed in Table 2, the correlation magnitude of Satellite PRN12 was a little smaller than that of Satellite PRN6 when acquired with the resampling strategy. Nevertheless, the correlation ratio of Satellite PRN12 was still the biggest among all acquired satellites. The acquisition results of the resampling strategy kept consistent with that of the conventional acquisition algorithm in Table 2. It proves that setting the correlation ratio as acquisition threshold can enhance the acquisition performance, especially for cases: (1) correlation magnitudes are too small in the search space due to noise in the received broadband signal; (2) there exists more than one value which is higher than the conventional correlation threshold (always a constant of correlation magnitude).

**Figure 8:**
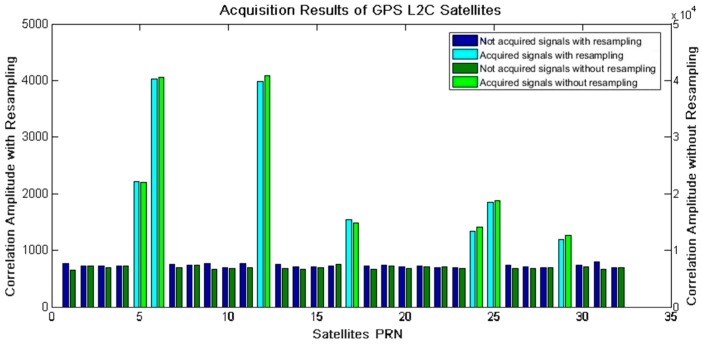
Acquisition results for GPS L2C satellites without/with the resampling strategy.

Satellites acquired by the resampling strategy and the conventional acquisition algorithm were completely the same, as illustrated in Figure 8. This has proved the effectiveness of the proposed resampling strategy for signal acquisition. Furthermore, for acquired (cyan and bright green bars) and not acquired (dark blue and blackish green bars) satellites, the correlation magnitude of the resampling strategy (the left *Y* axis) was about 10% that of the conventional one (the right *Y* axis), corresponding to results in Table 2.

**Figure 9:**
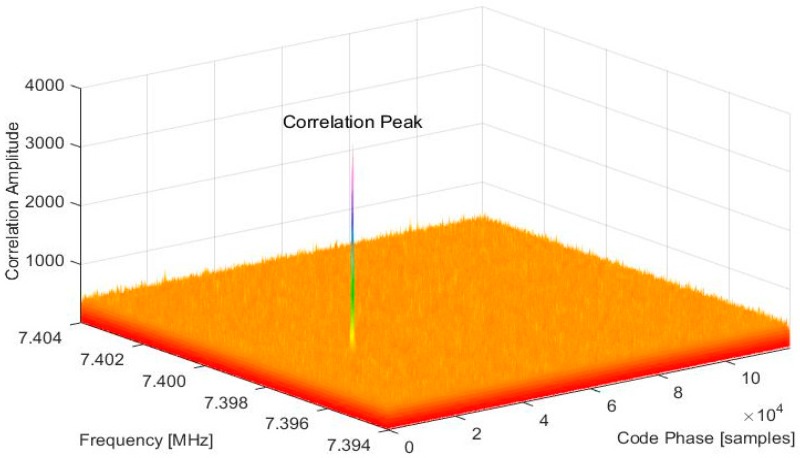
Correlation ratios of Satellite PRN12 acquired using the resampling strategy.

Doppler frequency shift, code phase offset, and correlation ratio of acquired satellites in Table 2 can be demonstrated clearly in the search space of frequency and code phase, for instance, the acquisition result of Satellite PRN12 shown in Figure 9. The correlation peak occurred at the point of frequency $\hat{f}=7.39853\;\mathrm{MHz}$ and code phase $\hat{\theta }=193394\;\left(\mathrm{samples}\right)$. The highest peak was much bigger ($R_{c}>7$) than other correlation magnitudes in the search space. Therefore, Satellite PRN 12 was acquired successfully, the carrier frequency is 7.39853 MHz and code phase is 0.365π (1866 chips). The acquisition results were adopted as estimated parameters for the subsequent signal tracking.

**Figure 10:**
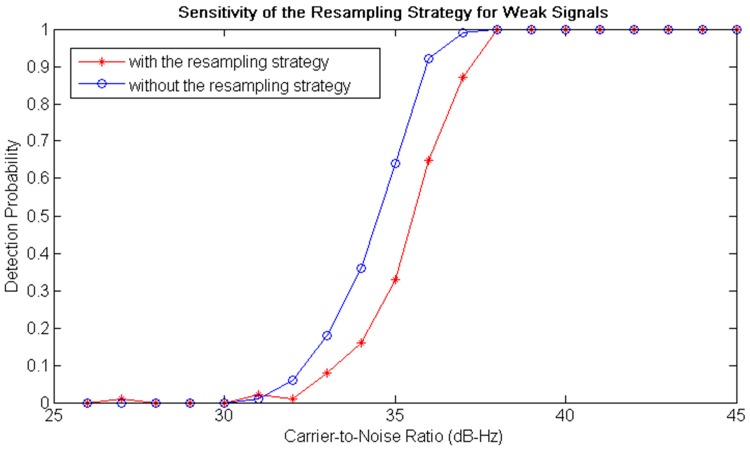
Sensitivity of the resampling strategy for weak signals. The red plot is the detection probability of signal acquisition with the resampling strategy, and the blue one is that of the conventional acquisition algorithm (without the resampling strategy).

Aimed at investigating the sensitivity of the resampling strategy under weak signal conditions, extensive Monte Carlo simulations were performed by employing a high-confidence software IF signal simulator [38]. The carrier-to-noise ratio was set to vary from *25* to 45 dB-Hz with a step of 1 dB-Hz and circular correlation time of signal acquisition was set as $L_{x}=20\;\mathrm{ms}$. The experiments of signal acquisition were repeated for 200 times for each carrier-to-noise ratio. Detection probabilities are evaluated and compared for signal acquisition without/with the resampling strategy. From the experimental results of sensitivity illustrated in Figure 10, it can be found that the signal could be acquired successfully with the resampling strategy when the carrier-to-noise ratio was bigger than 32 dB-Hz, and detection probability had reached 100% with the carrier-to-noise ratio of 38 dB-Hz. Compared to the detection probability without the resampling strategy (the blue plot), the sensitivity of the resampling strategy had a loss of nearly 1 dB. It can be attributed to the bandpass sampling filter that filters out some powers of the side lobe signals from the received broadband signal. However, the significant computational cost reduction obtained can make up for the sensitivity loss, as longer circular correlation time and non-coherent integration can be adopted to increase the sensitivity.

**Figure 11:**
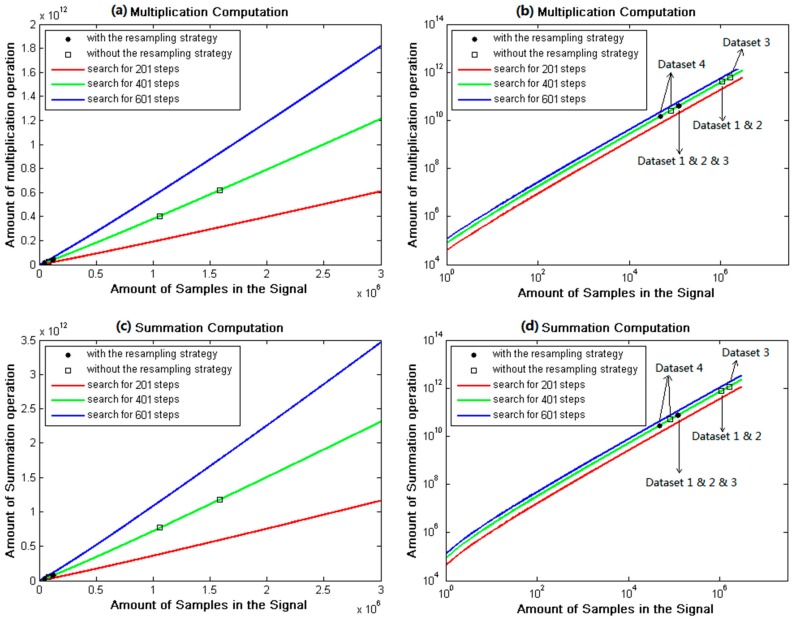
Computation of signal acquisition without/with the resampling strategy for all experimental datasets: (**a**) Multiplication computation in linear axis; (**b**) Multiplication computation in log axis; (**c**) Summation computation in linear axis; (**d**) Summation computation in log axis.

In order to validate the effects of the proposed strategy on computation and time cost of signal acquisition, comparative experiments without/with the resampling strategy were conducted on all the datasets. Experimental results are presented in Table 3 and Figure 11. Meanwhile, the circular correlation time was fixed as $L_{x}=20\;\mathrm{ms}$ to eliminate the effects of variable circular correlation time. With respect to the comparison in Table 3, the resampling frequency was reduced to about 10% of the conventional sampling frequency by applying the resampling strategy to Dataset 1, 2, and 3. In Figure 11, computations (multiplication and summation operations) of all the datasets are linearly related to the amount of frequency bins and the data size of the sampled signal. The amount of frequency bins was set as a constant, based on the width of frequency search range and the bandwidth of each frequency bin. And the data size of the sampled signal was exactly determined by the resampling frequency. Resulting from the reduced sampling frequency, the computation cost of signal acquisition with the resampling strategy was greatly decreased, nearly 90–94% of that without the proposed strategy, as shown in Table 3 and Figure 11. Besides, the received broadband signal in Dataset 4 was an 8-bit complex signal with negative intermediate frequency. The bandpass filter of the proposed resampling strategy was removed since it was not applicable to extract the main lobe signal of Dataset 4. Additionally, the conventional sampling frequency of Dataset 4 was low enough. Thus, the resampling frequency reduced to about 59%, not as nearly 10% for other datasets. Correspondingly, the computation reduction of 43% was gained by applying the resampling strategy to signal acquisition of Dataset 4. Meanwhile, for all experimental datasets, the time cost of signal acquisition with the resampling strategy reduced to about 7.7~65% of that with the conventional acquisition algorithm, accompanying with the reduced computation.

Experimental results in Table 3 and Figure 11 prove that the adoption of the resampling strategy directly resulted in the significant reduction on the resampled signals, which were used for FFT, correlation, and convolution operations. It makes the dominated contribution to the improved performance of computation and time cost of signal acquisition. Moreover, it is concluded that the resampling strategy can demonstrate better performance for the received broadband signal of higher conventional sampling frequency.

### 4.3. Performance of Variable Circular Correlation Time

Variable circular correlation times are adopted for the coarse signal acquisition of all the datasets in the experiments to explore the relationship between circular correlation time and time cost of signal acquisition, as well as acquisition effectiveness.

**Figure 12:**
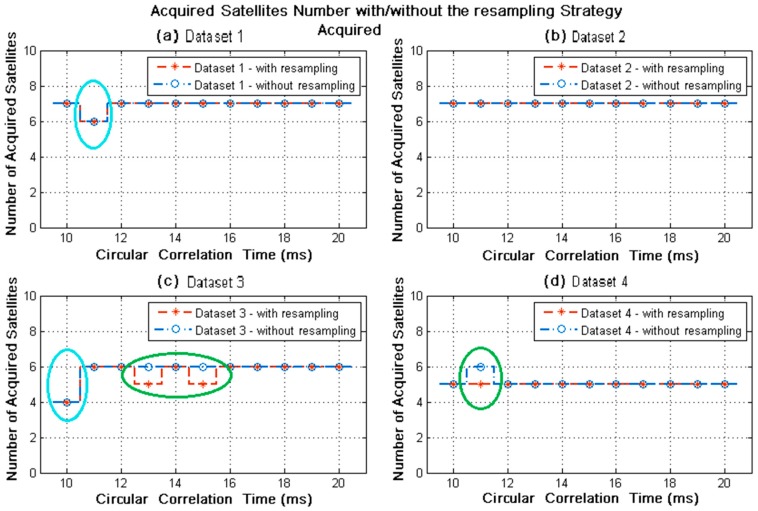
Number of satellites acquired without/with the resampling strategy for variable circular correlation time. The green circles indicate the difference of acquisition results without/with the resampling strategy; the cyan circles indicate the incomplete acquisition results with too short circular correlation time.

For all the datasets, the number of satellites acquired without/with the resampling strategy is shown in Figure 12 when circular correlation time was varying from 10 ms to 20 ms. The experimental results are exhibited as the comparative red and blue plots to illustrate the effects of the resampling strategy. There are several cases, marked as green circles in Figure 12c,d, in which the number of satellites acquired with the resampling strategy was fewer than that with the conventional acquisition algorithm. However, there were still enough acquired satellites for navigation solution in these cases. Except that, the majority of experimental results with the resampling strategy were the same with that of the conventional one. This means that acquisition effectiveness of the resampling strategy can primarily reach that of the conventional acquisition algorithm, which cost about 10 times computation and time of the resampling strategy. Further, the number of satellites acquired without/with the resampling strategy nearly remained unchanged when circular correlation time was increasing from 12 ms to 20 ms. This indicates that there is potential to shorten the circular correlation time so as to reduce computation and time cost, without obvious loss of acquisition performance. Whereas, too short circular correlation time reasonably leads to the incomplete acquisition of visible satellites, as cyan circles in Figure 12a,c.

**Figure 13:**
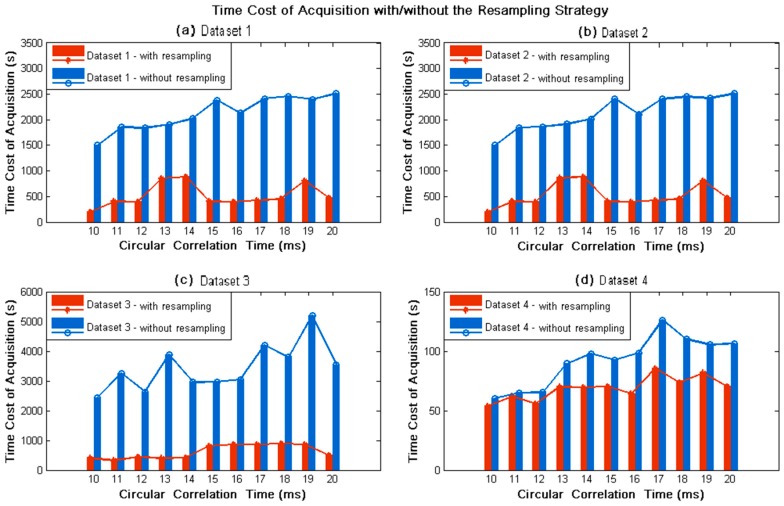
Time cost of signal acquisition with/without the resampling strategy for variable circular correlation time. Red bars and plot represent acquisition results with the resampling strategy, while blue ones are for that of the conventional acquisition algorithm.

The time cost performance of variable circular correlation time on all experimental datasets is presented in Figure 13, when acquiring without/with the resampling strategy. Noticeably, when circular correlation time was varying from 10 ms to 20 ms, the time cost of signal acquisition with the resampling strategy (red bars) were much less than that of the conventional algorithm (blue bars), nearly decreased by 60–90% for Datasets 1, 2, and 3, while 5–35% for Dataset 4. This is attributed to the fact that the bandpass filter of the resampling strategy is not suitable for the signal with negative intermediate frequency in Dataset 4. The experimental results in Figure 13 illustrate the capability of the resampling strategy to decrease the time cost of signal acquisition. In addition, the blue and red plots in Figure 13 reflected the changes of time cost when the circular correlation time was varying. The time cost of signal acquisition without the resampling strategy seemed to increase plainly. Nevertheless, by linearly fitting these values, the time cost exhibited an increase of 3.6%, 3.6%, 3.8%, and 4.7% per millisecond for the 4 datasets, respectively, when the circular correlation time was varying from 10 ms to 20 ms. By contrast, the time cost for the resampling strategy looked stable, despite the existence of some outliers. Actually, the time cost of signal acquisition increased by 2.7%,2.7%,5.7%, and 2.7% per millisecond for the 4 datasets, respectively.

These experimental results indicate that a relatively shorter circular correlation time is beneficial for signal acquisition with both the proposed resampling strategy and the conventional acquisition algorithm, leading to less computation and time cost without obvious loss of acquisition performance.

## 5. Conclusions

The resampling strategy and variable circular correlation time are proposed to decrease computational complexity and time consumption of signal acquisition for GNSS receivers. The resampling strategy is inspired by the bandpass sampling theory and is applicable for broadband multi-frequency signals of widely-used multimode multiband GNSS receivers. The realization of the proposed resampling strategy is described in detail for combination with conventional acquisition algorithms. Specific to the much longer code period of satellite signals, variable circular correlation time is investigated to explore the potential of shortening the circular correlation time without obvious loss of acquisition performance. Moreover, the signal of the pilot channel is utilized to acquire the high-accuracy carrier frequency in the fine acquisition process. The acquisition threshold is set as the ratio of the highest and second highest correlation results in the search space of carrier frequency and code phase. Besides, the sensitivity of the resampling strategy to weak signals is investigated by extensive Monte Carlo simulations, and the computational complexity of signal acquisition is formulated by amounts of multiplication and summation operations to evaluate the efficiency of the signal acquisition algorithms.

Four sets of real GPS L2C signals are applied by comparative experiments to verify the effectiveness and efficiency of the proposed resampling strategy and variable circular correlation time. It has been proven that the resampling strategy has effectively decreased the computation and time cost of signal acquisition by nearly 90–94% without obvious loss of acquisition performance, although the sensitivity of the resampling strategy had a loss of nearly 1 dB and detection probability reached 100% when carrier-to-noise ratio was up to 38 dB-Hz. The greater decrease is achieved for the received broadband signal with the higher intermediate frequency and conventional sampling frequency. Besides, with circular correlation time varying from 10 ms to 20 ms, the time cost of signal acquisition increased by about 2.7–5.6% per millisecond, and the number of acquired satellites has no obvious changes for the cases with the resampling strategy and the conventional acquisition algorithm.

## Figures and Tables

**Table 1:** The main parameters of GNSS signals used in the experiments.

Dataset No.	1	2	3	4
Data Type	8-bit Real Data	8-bit Real Data	8-bit Real Data	8-bit Complex Data
Intermediate Frequency (MHz)	7.4	7.4	7.6	−0.02
Conventional Sampling Frequency (MHz)	53	53	79.25	4

**Table 2:** Acquisition results of the received signal in Dataset 1 without/with the resampling strategy.

Acquired Satellites		Acquisition without the Resampling Strategy					Acquisition with the Resampling Strategy				
PRN	CNo (dB-Hz)	Frequency	Doppler	Code Phase	Magnitude	Ratio	Frequency	Doppler	Code Phase	Magnitude	Ratio
		(MHz)	(Hz)	(samples)	/	/	(MHz)	(Hz)	(samples)	/	/
5	38.0	7.39649	−3507	701962	**21976**	3.9492	7.39649	−3508	701963	**2210**	3.9103
6	42.8	7.40103	1032	356345	**40595**	**6.8311**	7.40103	1033	356347	**4033**	**6.2393**
12	43.1	7.39853	−1470	193389	**40928**	**7.0512**	7.39853	−1465	193394	**3983**	**7.2284**
17	33.7	7.40119	1187	879016	**14909**	2.6156	7.40119	1186	879013	**1546**	2.6421
24	33.2	7.40152	1522	829115	**14041**	2.6483	7.40152	1522	829119	**1342**	2.2496
25	35.9	7.39697	−3027	621138	**18820**	3.4106	7.39697	−3030	621140	**1843**	3.2997
29	27.6	7.39708	−2920	933174	**12710**	2.1654	7.39708	−2922	933179	**1196**	2.1817

**Table 3:** Sampling frequency, computation, and time cost of signal acquisition without/with the resampling strategy for the received broadband signals of different experimental datasets.

DataSet No.	Acquisition without the Resampling Strategy			Acquisition with the Resampling Strategy		
	Sampling Frequency (MHz)	Computation ($O^{M},\;O^{A}$)	Time Cost (s)	Sampling Frequency (MHz)	Computation ($O^{M},\;O^{A}$)	Time Cost (s)
1	53.00	(6.8 × 10^11^, 1.3 × 10^12^)	2486	5.97	(6.7 × 10^10^, 1.3 × 10^11^)	246.8
2	53.00	(6.8 × 10^11^, 1.3 × 10^12^)	2467	5.97	(6.7 × 10^10^, 1.3 × 10^11^)	244.9
3	79.25	(1.1 × 10^12^, 2.0 × 10^12^)	3455	6.13	(6.9 × 10^10^, 1.3 × 10^11^)	268.5
4	4.00	(4.4 × 10^10^, 8.2 × 10^11^)	109.5	2.36	(2.5 × 10^10^, 4.7 × 10^11^)	70.2
